# Structure activity relationship (SAR) and quantitative structure activity relationship (QSAR) studies showed plant flavonoids as potential inhibitors of dengue NS2B-NS3 protease

**DOI:** 10.1186/s12900-018-0084-5

**Published:** 2018-04-19

**Authors:** Muhammad Waseem Sarwar, Adeel Riaz, Syed Muhammad Raihan Dilshad, Ahmed Al-Qahtani, Muhammad Shah Nawaz-Ul-Rehman, Muhammad Mubin

**Affiliations:** 1 Virology Lab, Centre of Agricultural Biochemistry and Biotechnology, University of Agriculture, Jail road, Faisalabad, 38000 Pakistan; 2 Department of Infection and Immunity, Research Center, King Faisal Specialist Hospital and Research Center, Riyadh, Saudi Arabia; 3 Department of Microbiology and Immunology, College of Medicine, Alfaisal University, Riyadh, Saudi Arabia; 4 Liver Disease Research Center, King Saud University, Riyadh, Saudi Arabia; 5 Department of Theriogenology, Faculty of Veterinary and Animal Sciences, Gomal University, Dera Ismail Khan, Pakistan

**Keywords:** Flavonoids, QSAR, SAR, Molecular docking, Dengue virus, NS2B-NS3

## Abstract

**Background:**

Due to dengue virus disease, half of the world population is at severe health risk. Viral encoded NS2B-NS3 protease complex causes cleavage in the nonstructural region of the viral polyprotein. The cleavage is essentially required for fully functional viral protein. It has already been reported that if function of NS2B-NS3 complex is disrupted, viral replication is inhibited. Therefore, the NS2B-NS3 is a well-characterized target for designing antiviral drug.

**Results:**

In this study docking analysis was performed with active site of dengue NS2B-NS3 protein with selected plant flavonoids. More than 100 flavonoids were used for docking analysis. On the basis of docking results 10 flavonoids might be considered as the best inhibitors of NS2B-NS3 protein. The interaction studies showed resilient interactions between ligand and receptor atoms. Furthermore, QSAR and SAR studies were conducted on the basis of NS2B-NS3 protease complex docking results. The value of correlation coefficient (*r*) 0.95 shows that there was a good correlation between flavonoid structures and selected properties.

**Conclusion:**

We hereby suggest that plant flavonoids could be used as potent inhibitors of dengue NS2B-NS3 protein and can be used as antiviral agents against dengue virus. Out of more than hundred plant flavonoids, ten flavonoid structures are presented in this study. On the basis of best docking results, QSAR and SAR studies were performed. These flavonoids can directly work as anti-dengue drug or with little modifications in their structures.

## Background

Dengue virus belonging to family *Flaviviridae* is the most prevalent arthropod transmitted virus in humans. It can cause symptoms ranging from self-limiting dengue fever to sometimes-fatal dengue hemorrhagic fever [1].

Dengue virus is a positive sense single stranded ssRNA virus with 10.7 kb genome. Viral RNA is translated into a single polyprotein. The poly protein is cleaved by virus encoded NS2B/NS3 protease and the host proteases into structural proteins C, M, and E as well as nonstructural proteins NS1, NS2A, NS2B, NS3, NS4A, NS4B, and NS5 to initiate the replication of dengue virus [2, 3].

**Fig. 1:**
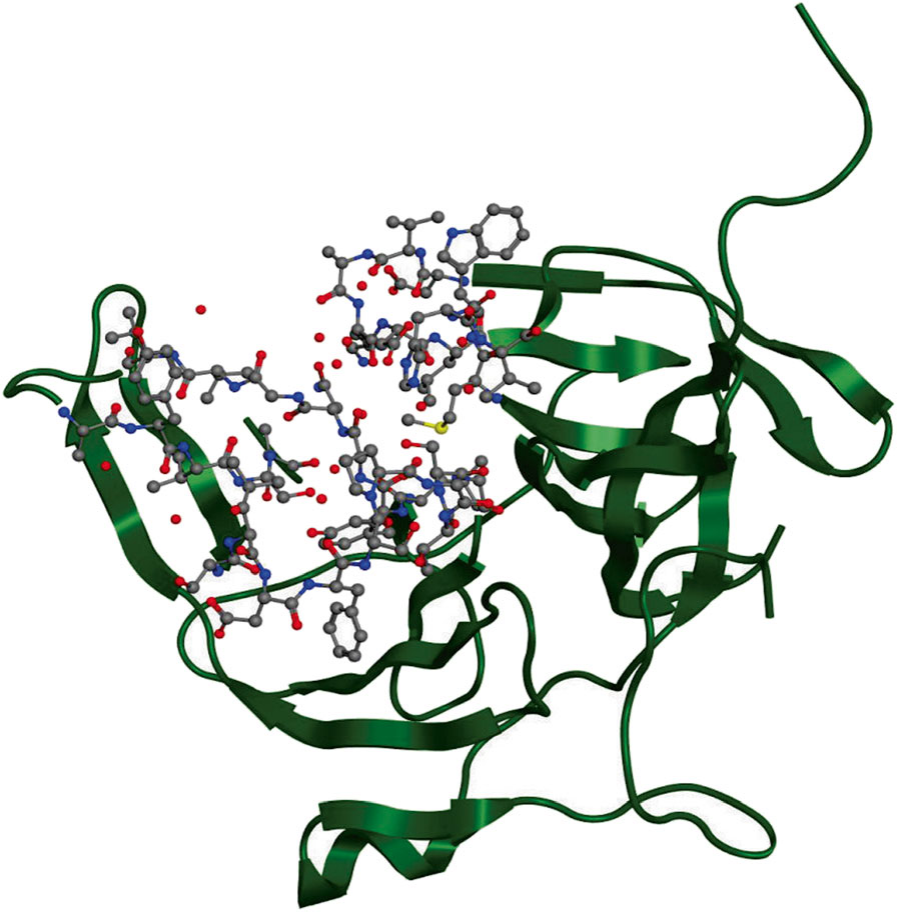
Structure of dengue NS2B-NS3 (2FOM); Catalytic site is shown in ball stick model

The NS2B-NS3 protease contains two functional regions i.e., a C-terminal region acting as RNA helicase and a N-terminal 180-residue is a trypsin like serine protease (Fig. 1). NS3 protease requires the central hydrophilic region of NS2B (NS2B; residues 49 to 95) to perform proteolytic activity and to stabilize folding. Thus, hydrophilic domain of NS2B interacts with NS3 protease and forms full active site [4]. The activity of NS2B/NS3 is critical for viral replication [5] as the disruption of NS2B-NS3 function inhibits viral replication [6–8]. So NS2B/NS3 protease could be targeted for the development of anti-DENV inhibitors.

Plants had served as a source of medicinal compounds for a long time and are basis of many pharmaceuticals now days [9]. Flavonoids are plant based phenolic compounds [10] having various biological properties like antiviral [11, 12], antioxidant, antifungal [13], anti-cancerous [14, 15], anti-angiogenic [16] and anti-inflammatory properties [17, 18].

Henceforth, flavonoids may act as inhibitors of dengue NS2B-NS3. In this study, *in-silico* screening using automated docking method was performed and binding models of dengue NS2B-NS3 protease with selected plant flavonoids are proposed. Finally, ten plant flavonoids were suggested as potential inhibitors of dengue virus NS2B-NS3 complex. Furthermore extensive studies of binding modes were performed using SAR model i.e., (Structure Activity Relationship) and QSAR model i.e., (Quantity Structure Activity Relationship) [19]. This study provides the novel insights in the development of anti-viral drugs against dengue virus.

## Methods

All analyses presented here were performed using 64-bit Operating System and Intel(R) Core(TM) **i5**-5200 U processor with 2.2 GHz processing speed. MOE (Molecular Operating Environment) software was used for computational analysis, provided by chemical computing group Inc. and Chimera software was used for protein structure manipulation.

### Preparation of receptor structure

Crystal structure of NS3-NS2B protease was obtained from Protein Data Bank (http://www.rcsb.org) with PDB ID 2FOM [20]. The protein consists of two chains and 185 residues length with resolution 1.5 Å. The ribbon diagram of target structure with catalytic site is shown in Fig. 1. This structure was subjected to 3D protonation and energy minimization using parameters like (gradient: 0.05, Force Field: MMFF94X + Solvation) using MOE Program. For docking the minimized structure was used as the receptor protein [21].

### Ligand preparation

More than 100 chemical structures of ligand flavonoid molecules were downloaded online from chebi (http://www.ebi.ac.uk/chebi/) in .mol format. These structures were prepared for docking in LigX module of MOE program with parameters (gradient: 0.05, Force Field: MMFF94X).

### Docking setup and run

The binding sites for the target protein were calculated, for docking analysis, by MOE site finder and then confirmed with the binding site reported in literature. During docking setup, only this binding site (His51, Asp75 and Ser135) was used (Fig. 1) to find the correct conformation of the ligand. To bind the selected ligands with receptor protein, MOE docking program with default parameters was used. MOE London dG scoring function was used to estimate free energy of binding for each ligand from a given pose [22]. The functional form of London dG scoring function is a sum of terms:

$$ \Delta G=c+{E}_{flex}+\sum \limits_{h- bonds}{c}_{HB}{f}_{HB}+\sum \limits_{m- lig}{c}_M{f}_M+\sum \limits_{atoms\kern0.24em i}\Delta {D}_i $$

The difference in desolvation energies is calculated according to the formula [23].

$$ \Delta {D}_i={c}_i{R_i}^3\left\{\underset{u\notin A\cup B}{\iiint }{\left|u\right|}^{-6} du-\underset{u\notin B}{\iiint }{\left|u\right|}^{-6} du\right\} $$

The final docking output was saved for further investigations.

### Ligand interactions studies

The ligand interactions were studied by Ligand Interaction module in MOE Program. It includes 2D and 3D representations of ligand and receptor protein interactions and calculated distances among ligand and protein interacting atoms.

### Calculation of descriptors

Descriptor refers to the two-dimensional (2-D) or three-dimensional (3-D) physiochemical property of a molecule [23]. The descriptors that were calculated in this study are as follows. logP(o/w) is the logarithm of the octanol and water partition coefficient. b_count is the number of bonds (including implicit hydrogens). This is calculated by the addition of (di/2 + hi) over all nontrivial atoms i. b-rotN is the number of rotatable bonds. With order 1, having at least two heavy neighbors and provided it is not in a ring, a bond is said to be rotatable. chi1 is the atomic connectivity index. a_acc is the number of hydrogen bonds of acceptor atoms (not counting acidic atoms but counting atoms that are both hydrogen bond donors and acceptors such as -OH). Q_pc + is the total positive partial charge i.e., the sum of the positive qi. Q_PC+ is identical to PC+ that has been reserved for compatibility. Q_pc-PC is the total negative partial charge and it is the sum of the all negative qi. SlogP is the logarithm of the octanol and water partition coefficient (including hydrogens). Log S is the logarithm of the aqueous solubility (mol/L). apol is the sum of the atomic polarizabilities. a_don is the number of hydrogen bond acceptor atoms. vsurf_g is the surface globularity. vsurf_wpu is the Hydrophilic volume. E is the value of the potential energy. E_ele is the electrostatic component of the potential energy. E_nb is the value of the potential energy with all bonded positions that have been disabled.

### Studies about structure activity relationship (SAR) and quantitative structure activity relationship (QSAR)

The Structure-Activity Relationship (SAR) report was generated by MOE application to find common scaffolds in flavonoid structures used in this study. The input data was flavonoid structures in .mol file format. Structure-Activity Report involves gathering of input data (molecules, activity, predefined scaffolds, etc.) and detection of common scaffolds. After that, Alignment of the scaffolds was done to produce a common numbering system by using ligand alignment module of MOE program.

The Quantitative Structure Activity Relationship (QSAR) was done in MOE. All docked molecules were used for training set. In a test set, QSAR model correlates the activities with properties inherent to each molecule. Different molecular descriptors were used to evaluate these properties. QSAR studies involve two steps. In first step, descriptors were generated that encode chemical structure information. During second step, a statistical regression technique is employed to correlate the structural variation, as encoded by the descriptors, with the variation in the biological activity of protein. To test the reliability of results, regression analysis was performed using inhibitory activity as dependent variable and the descriptor as predictor variables. After making sure the reasonable correlation of inhibitory activity with the individual descriptor, QSAR models were derived The predictor variables with *p* value greater than 0.05 were eliminated while obtaining the QSAR models, to assure their statistical reliability.

## Results

### Docking analysis

**Fig. 2:**
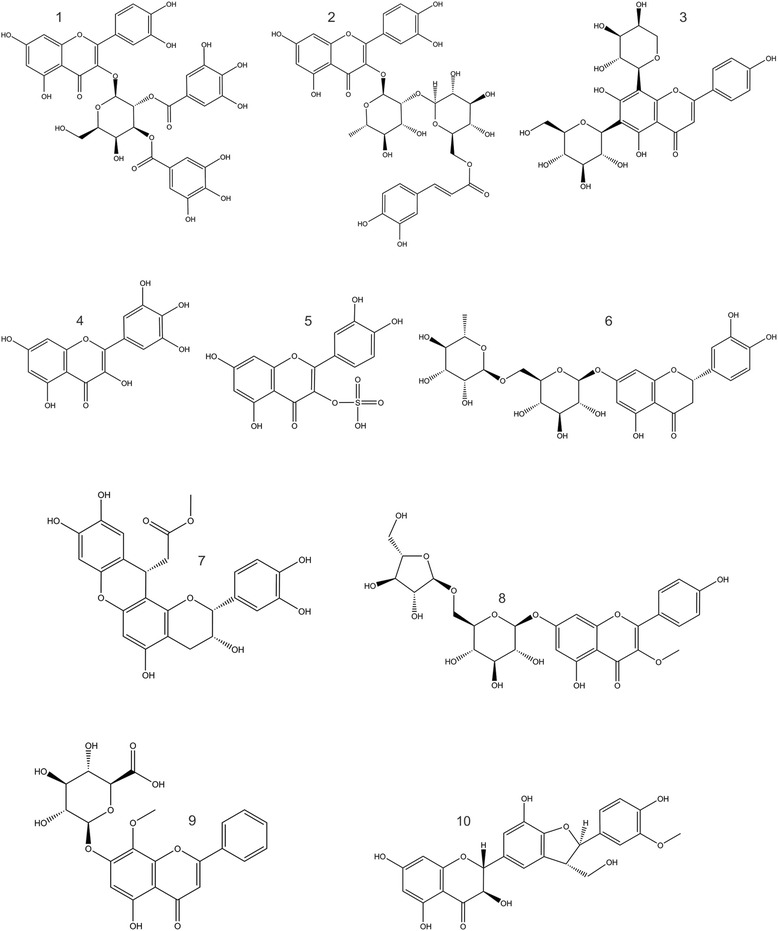
Flavonoid structures that shown best results of docking analysis; 1. quercetin 3-O-(2″,3″-digalloyl)-β-D-galactopyranoside 2. quercetin 3-O-α-(6″‘-caffeoylglucosyl-β-1,2-rhamnoside) 3. schaftoside 4. myricetin 5. quercetin 3-sulfate 6. eriocitrin 7. catiguanin B 8. 4′,5,7-trihydroxy-3-methoxyflavone-7-O-α-L-arabinofuranosyl(1 → 6)-β-D-glucopyranoside 9. wogonin 7-O-β-D-glucuronide 10. silychristin

Docking of all flavonoid structures (Fig. 2) was done against the active site of dengue NS2B-NS3 protein. Docking analysis provided a number of configurations that were scored to determine favorable binding modes. The flavonoid structures with high docking scores with molecular data are summarized in Table 1.

**Table 1:** Selected flavonoid molecules downloaded from chebi (http://www.ebi.ac.uk/chebi/), shown higher docking score

Flavonoid No.	Chebi ID	Name	Plant Sources	Docking Score (Kcal/mol)
1	66,287	quercetin 3-O-(2″,3″-digalloyl)-β-D-galactopyranoside	Euphorbia lunulata	− 26.101
2	66,284	quercetin 3-O-α-(6″‘-caffeoylglucosyl-β-1,2-rhamnoside)	*Sedum sarmentosum*	−24.987
3	9047	schaftoside	*Passiflora tripartita*	−23.399
4	18,152	myricetin	*Myrica rubra*	−21.987
5	17,730	quercetin 3-sulfate	*Anethum graveolens*	−20.989
6	28,709	eriocitrin	Citrus lumia	−20.693
			Cyclopia subternata	
7	65,602	catiguanin B	Trichilia catigua	−20.414
8	68,348	4′,5,7-trihydroxy-3-methoxyflavone-7-O-α-L-arabinofuranosyl(1 → 6)-β-D-glucopyranoside	Lepisorus contortus	−20.378
9	61,282	wogonin 7-O-β-D-glucuronide	Scutellaria baicalensis	−20.102
10	9143	silychristin	*Silybum marianum*	−20.085

### Calculation of ligand interaction

**Fig. 3:**
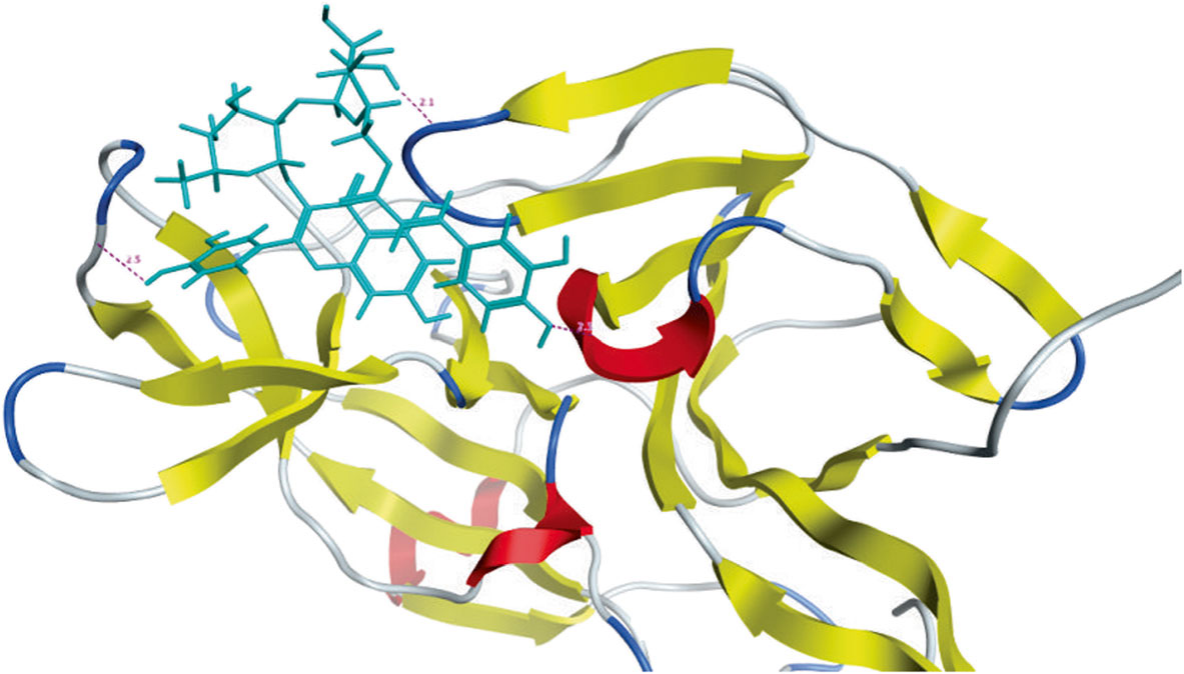
3D interaction diagram; Dotted lines showing the interactions between ligand and receptor protein atoms with calculated distances

**Fig. 4:**
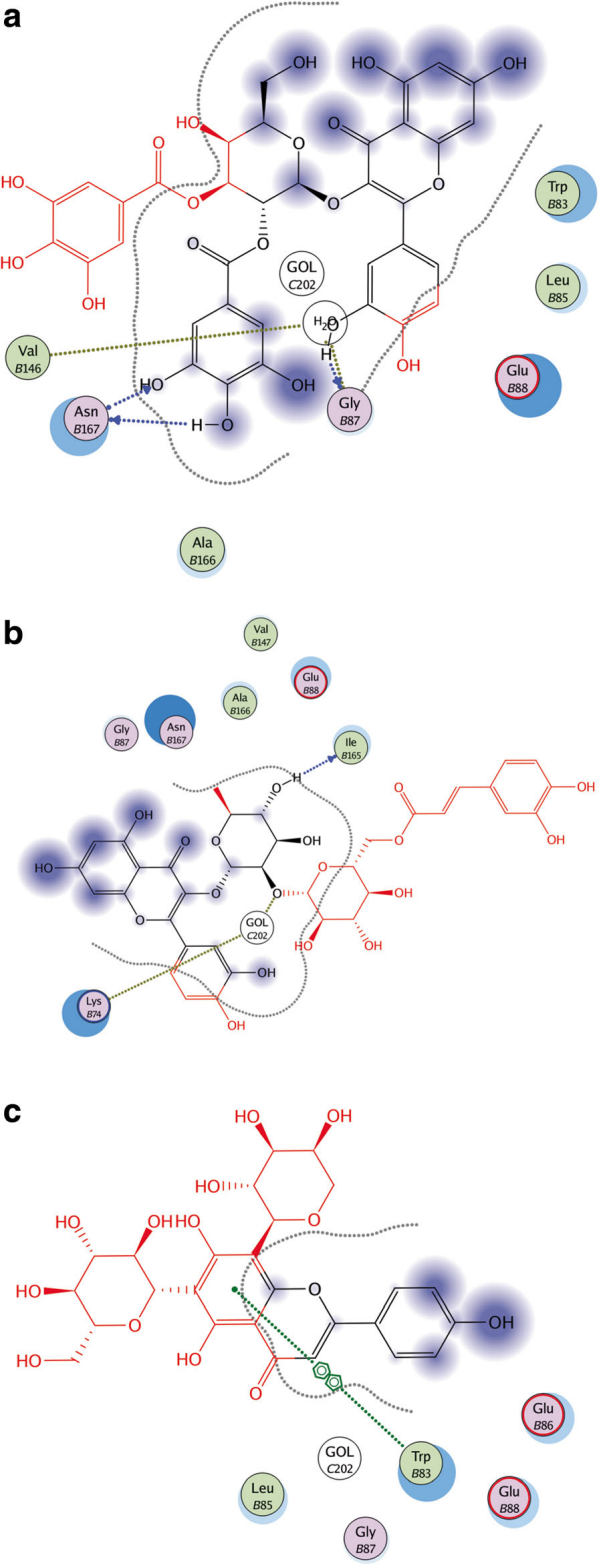
2D interaction diagram; showing the interacting ligand atoms with specific residues in receptor protein. Dotted lines are showing specific interaction. **a.** Flavonoid No.1 **b.** Flavonoid No. 2 **c.** Flavonoid No. 3

**Fig. 5:**
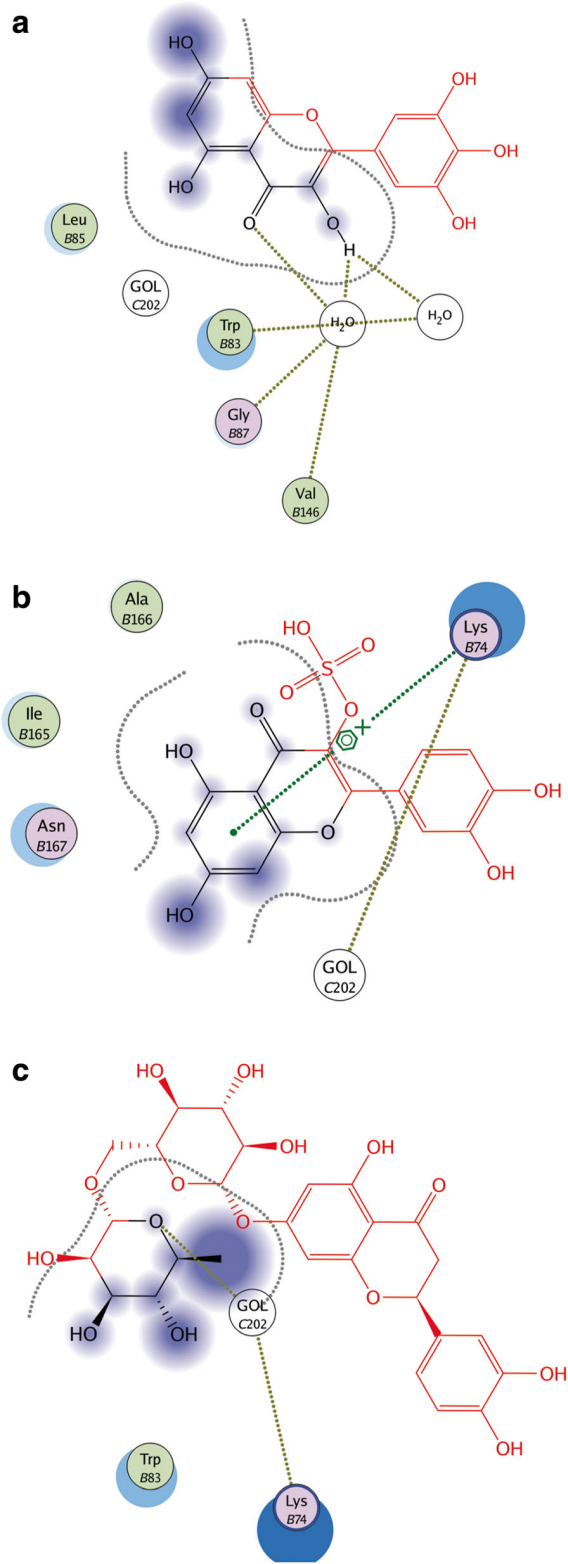
2D interaction diagram; showing the interacting ligand atoms with specific residues in receptor protein. Dotted lines are showing specific interaction. **a.** Flavonoid No. 4 **b.** Flavonoid No. 5 **c.** Flavonoid No. 6

**Fig. 6:**
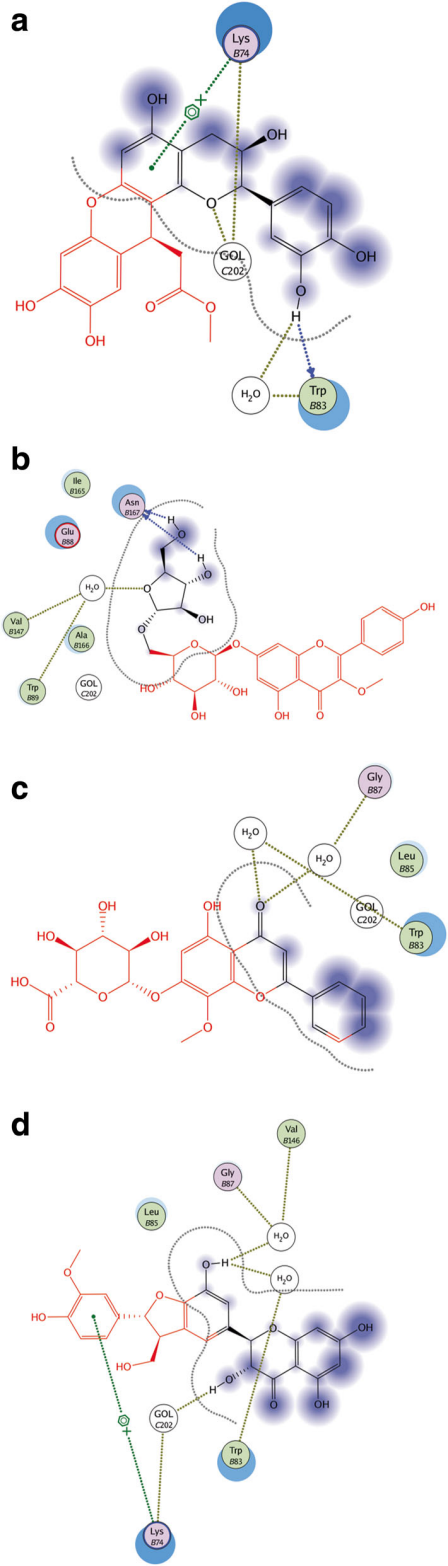
2D interaction diagram; showing the interacting ligand atoms with specific residues in receptor protein. Dotted lines are showing specific interaction. **a.** Flavonoid No.7 **b.** Flavonoid No. 8 **c.** Flavonoid No. 9 **d.** Flavonoid No. 10

Ligand interactions were obtained by MOE program. The Ligand Interactions studies are used to visualize an active site of a complex in diagrammatic form. Depicted view of 3D interaction is shown in Fig. 3. Flavonoid 1 formed an interaction with three amino acids of viral protein (Gly 87, Val 146 and Asn 167) with in the active site (Fig. 4a). Flavonid 2 formed the interaction with two amino acids of viral protein (Lys 74 and Ile 165) with in active site (Fig. 4b). Flavonoid 3 formed an arene-arene link with amino acid Trp 83 (Fig. 4c). Flavonoid 4 formed interaction with three amino acids (Trp 83 Gly 87 and Val 146) (Fig. 5a). Flavonoid 5 formed an arene cationic link with amino acid Lys 74 (Fig. 5b). Flavonoid 6 formed the interaction with Lys 74 (Fig. 5c). Flavonoid 7 interacted as a cationic arene interaction with Lys 74 and a hydrogen bond donation with Trp 83 (Fig. 6a). Flavonoid 8 formed interaction with Asn 167, Val 147 and Trp 89 (Fig. 6b). Flavonoid 9 has shown an interaction with amino acid Gly 87 and Trp 83 (Fig. 6c). Flavonoid 10 formed a cationic arene link with Lys 74 and an hydrogen bond with Trp 83 (Fig. 6d).

### Structure activity report

The Structure-Activity Report is a MOE application, which generates a web page housing an interactive view onto a collection of molecules, which are related by a small number of related common scaffolds. The application is designed to operate on small databases of drug-like molecules, which are in the process of being refined for improved binding affinity and other pharmacological properties. Report was generated using a panel from an interactive session of MOE, using a MOE molecular database. The default browser was launched to show the results, when the generation was completed.

**Fig. 7:**
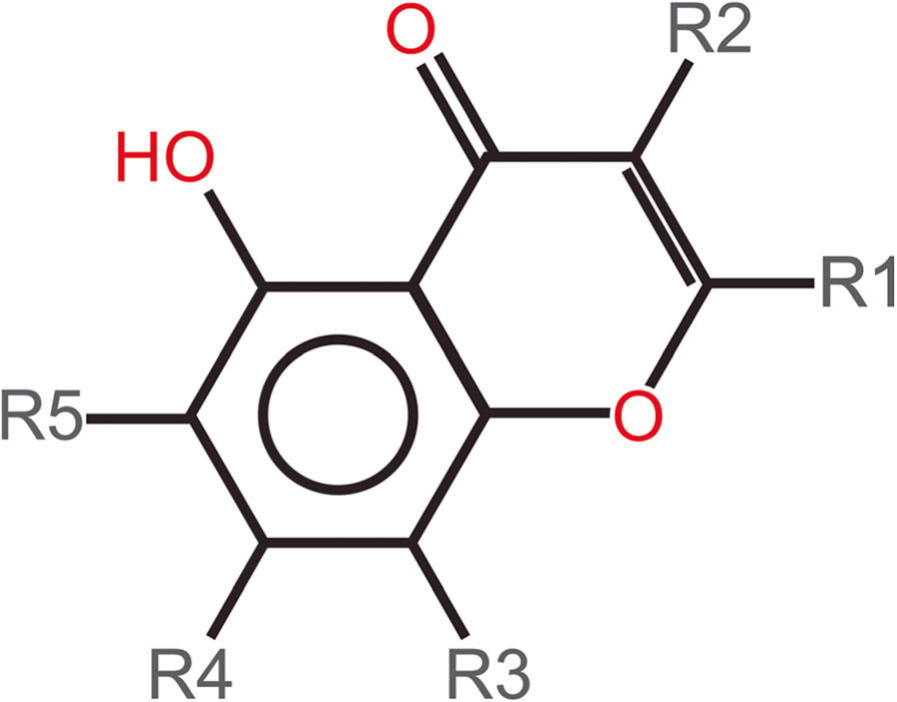
Common Scaffolds obtained from Structure Activity Report; **a.** Scaffold 1 common in sevenall flavonoid structures

Scaffold categorization needs a small number of common scaffolds for best results. Alignment of the scaffolds was done to produce a common numbering system by using ligand alignment module of MOE program. One common scaffold was obtained from the alignment of all ten flavonoid structures (Fig. 7). These results show that there is a prevalence of similarity among the structural formulas of different flavonoid compounds performing the same function i.e. binding with active site of NS3-NSB protein of dengue virus.

### QSAR model

**Fig. 8:**
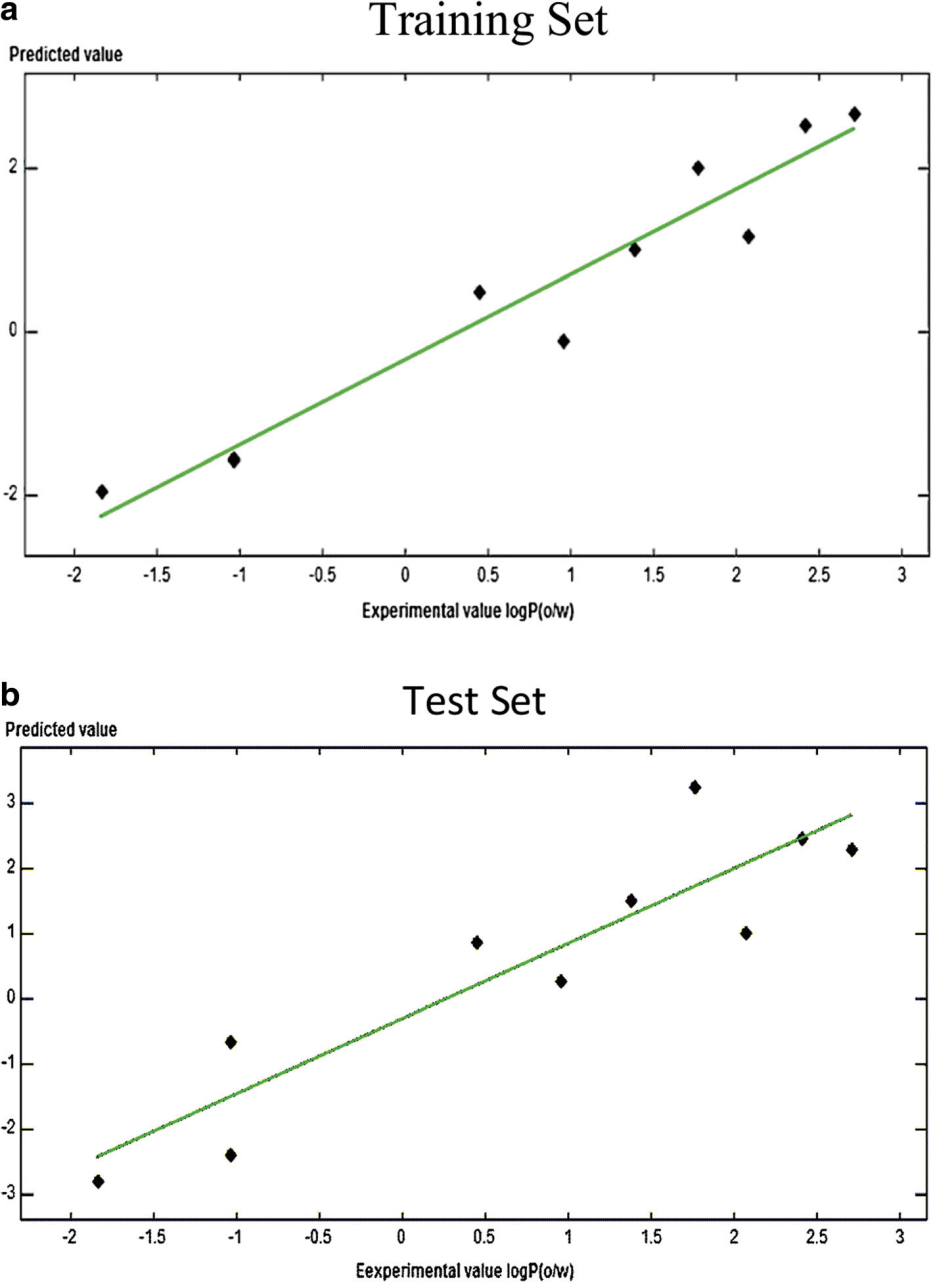
QSAR model; **a.** Training set **b.** Test set

The biochemical activities in this work were analyzed for cross correlation of activities and chemical descriptors to find out whether for observed inhibitory effect against NS2B-NS3 complex is there any common structure activity relationship. It was discovered that there was a high level of correlation between the observed parameters with a correlation coefficient (r) of value 0.956. So to clarify conceivable QSARs for each compound with its biological activity, atomic models of flavonoids were constructed. The structures of flavonoid compounds were shown in Fig. 2 and data was presented in Table 1. These flavonoids modeled keeping in view the patterns of hydrogen bonding and hydrophobic interactions and the induced fit phenomena that best express the binding affinity of each structure as accessed from logP values. These logP were separated as training set and test set values. Since there was no pervious information of flavonoid inhibition of dengue NS2B-NS3 complex available, 10 flavonoids showing best docking results were used for both training set as well as test set. The values of test set were approximately the same as that for training set (Fig. 8) clearly showing that there is a positive correlation between the values of logP and activities. The parameters used in QSAR model and the value of logP Correlation Co-efficient obtained are shown below

$$ {\displaystyle \begin{array}{l}\mathrm{Activity}\ \mathrm{field}=\mathrm{logP}\left(\mathrm{o}/\mathrm{w}\right),\mathrm{Descriptor}=\mathrm{SlogP},\mathrm{PLS}=\mathrm{RMSE}=0.24071,\mathrm{R}2=0.97662\\ {}\mathrm{logP}\ \mathrm{Correlation}\ \mathrm{Coeffient}=+0.77652,\mathrm{Normalization}=+0.95672\end{array}} $$

**Fig. 9:**
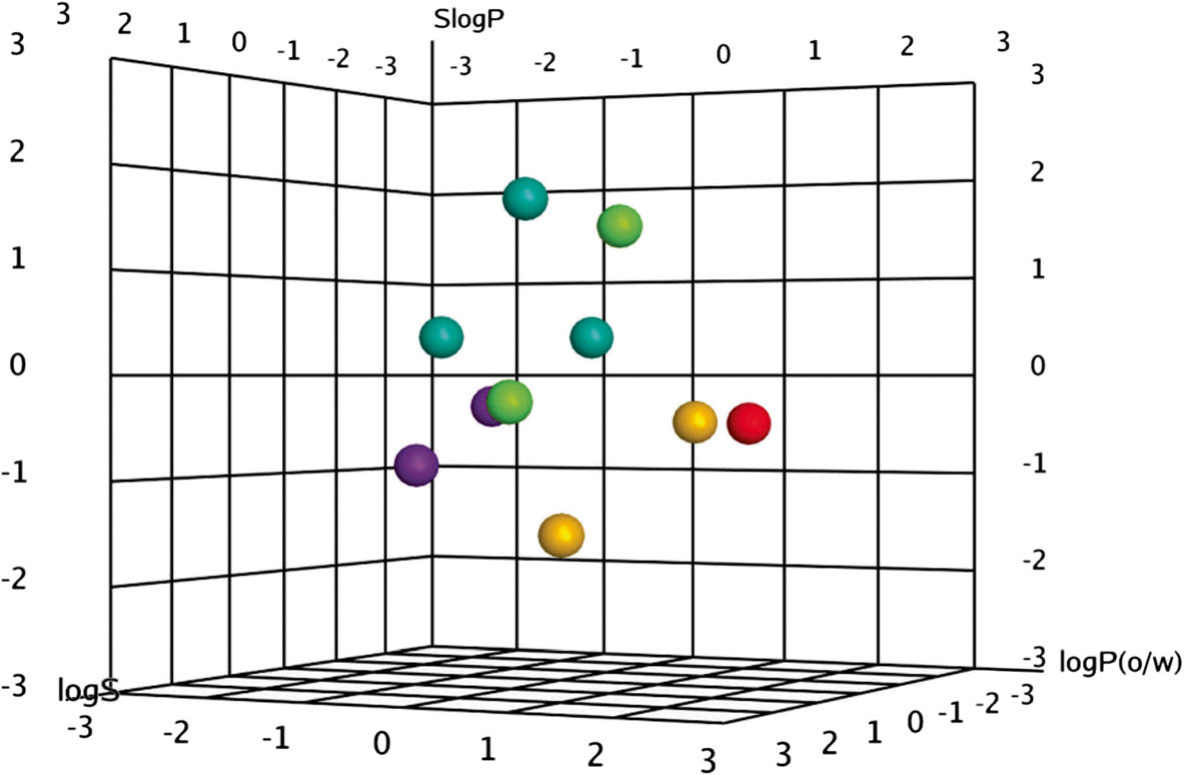
3D Correlation Plot for the activities of QSAR; X-axis logP(o/w), Y-axis SlogP, Z-axis logS

Accordingly partial least square (PLS) experiment was performed to achieve three dimensional-QSAR (Fig. 9). Leave one out statistical procedure was used to get ideal number of components. It was done to build a regression model that is statistically significant. Cross-validated coefficient q2 was used to measure the model quality. Conventional correlation coefficient r2 was utilized to obtain the external predictivity.

## Discussion

Dengue is a mosquito-borne viral hemorrhagic illness that is very dangerous to human wellbeing in tropical and subtropical areas. The virus-encoded protease activity, important for dengue virus infection, requires two viral proteins, NS2B and NS3 [24]. It has been already established that the disruption of NS2B-NS3 protein complex has negative effect on viral replication [25]. In the present investigation, plant flavonoids have been tested as potential inhibitor of NS2B-NS3 complex of viral protease. Previously, Plant flavonoids were known for anti-inflammatory, anticancer and antiviral activities [12, 14]. In present work we used structure based computational docking analysis tool to find the anti-dengue potential of flavonoids. More than 100 flavonoid structures were downloaded from chemebi. Docking of all flavonoid structures was done against the active site of dengue NS2B-NS3 protein. Docking analysis provided various designs that were scored to decide ideal restricting modes. We found very high scores of docking than previously reported data [26]. Docking analysis contributed towards basic idea and binding energy values for each ligand. After this, ligand interaction studies were done for the further confirmation of docking results and to have an insight into each interaction of atoms of ligand and protein. In our study we found more than one flavonoid as potential inhibitor of dengue NS2B-NS3 so it was necessary to find out the common structure among all those flavonoids that showed high binding score. As the compounds that have common or correlated structures have similar physicochemical properties and thus have similar binding modes and subsequently comparable biological activities. To find out a common structural feature among flavonoids, structure activity report was generated and a common conformation was obtained. We are reporting this conformation for very first time and it is an important step towards development of novel drugs against dengue virus. Quantitative structure activity relationships (QSAR) have been used for the development of relationships between physicochemical properties of compounds and their biological activities to get a statistical model, which prove their binding assays. The basic principle involved in this process is that the difference in the structure of chemical compounds is accountable for the variation in their biological activities [27]. In the present study the value of correlation obtained from QSAR model showed that there is linear relationship between selected calculated descriptor values and activities of compounds. We are reporting ten naturally occurring flavonoids as potential inhibitors of dengue NS2B-NS3 protein complex, which can inhibit viral replication. These flavonoids were not known previously for their anti-dengue activity.

## Conclusions

In present work, different plant flavonoids were docked and structure based SAR and QSAR analysis was done for dengue NS2B-NS3 protein. The results of statistical analysis of both models i.e., SAR and QSAR were convincing and comparable. The generated model was used to predict the effect of important structural characteristics of the potent inhibitor. The generated 3D-QSAR models were further verified by the docking studies of most potent and least active inhibitors so can be used for better drug development.

This study is an effort to explore novel inhibitors of dengue NS2B-NS3 and to through light on the structural and inhibitory activity of plant flavonoids. Consequently, ten flavonoids were found as inhibitors of NS2B-NS3. Henceforth, QSAR and SAR data provided in this study will serve as basis for a better drug development.

## Abbreviations

a_acc: Number of hydrogen bond acceptor atoms
apol: Sum of the atomic polarizabilities
Asp: Aspartic acid
b rotN: Number of rotatable bond
Chi1: Atomic connectivity index
DENV: Dengue virus
E_ele: Electrostatic component of the potential energy
E_nb: Value of the potential energy with all bonded term disabled
His: Histidine
Log P: Partition coefficient
MOE: Molecular operating environment
NS2B: Non structural protein 2B
NS3: Non structural protein 3
OPLS-AA: Optimized potentials for liquid simulations for amino acids
PLS: Partial least square analysis
Q_pc+: Total positive partial charge
QSAR: Quantitative structure activity relationship
R2: Conventional correlation coefficient
RMSE: Root mean square error
SAR: Structure activity relationship
Ser: Serine
SlogP: Log of the octanol/water partition coefficient
vsurf_g: Surface globularity
vsurf_wpu: Hydrophilic volume
